# Potential distribution of mosquito vector species in a primary malaria endemic region of Colombia

**DOI:** 10.1371/journal.pone.0179093

**Published:** 2017-06-08

**Authors:** Mariano Altamiranda-Saavedra, Sair Arboleda, Juan L. Parra, A. Townsend Peterson, Margarita M. Correa

**Affiliations:** 1 Grupo de Microbiología Molecular, Escuela de Microbiología, Universidad de Antioquia, Medellín, Colombia; 2 Grupo Biología y Control de Enfermedades Infecciosas, Universidad de Antioquia, Medellín, Colombia; 3 Grupo de Ecología y Evolución de Vertebrados, Instituto de Biología, Universidad de Antioquia, Medellín, Colombia; 4 Biodiversity Institute, University of Kansas, Lawrence, Kansas, United States of America; Swedish University of Agricultural Sciences, SWEDEN

## Abstract

Rapid transformation of natural ecosystems changes ecological conditions for important human disease vector species; therefore, an essential task is to identify and understand the variables that shape distributions of these species to optimize efforts toward control and mitigation. Ecological niche modeling was used to estimate the potential distribution and to assess hypotheses of niche similarity among the three main malaria vector species in northern Colombia: *Anopheles nuneztovari*, *An*. *albimanus*, and *An*. *darlingi*. Georeferenced point collection data and remotely sensed, fine-resolution satellite imagery were integrated across the Urabá –Bajo Cauca–Alto Sinú malaria endemic area using a maximum entropy algorithm. Results showed that *An*. *nuneztovari* has the widest geographic distribution, occupying almost the entire study region; this niche breadth is probably related to the ability of this species to colonize both, natural and disturbed environments. The model for *An*. *darlingi* showed that most suitable localities for this species in Bajo Cauca were along the Cauca and Nechí river. The riparian ecosystems in this region and the potential for rapid adaptation by this species to novel environments, may favor the establishment of populations of this species. Apparently, the three main Colombian *Anopheles* vector species in this endemic area do not occupy environments either with high seasonality, or with low seasonality and high NDVI values. Estimated overlap in geographic space between *An*. *nuneztovari* and *An*. *albimanus* indicated broad spatial and environmental similarity between these species. *An*. *nuneztovari* has a broader niche and potential distribution. Dispersal ability of these species and their ability to occupy diverse environmental situations may facilitate sympatry across many environmental and geographic contexts. These model results may be useful for the design and implementation of malaria species-specific vector control interventions optimized for this important malaria region.

## Introduction

**Fig 1:**
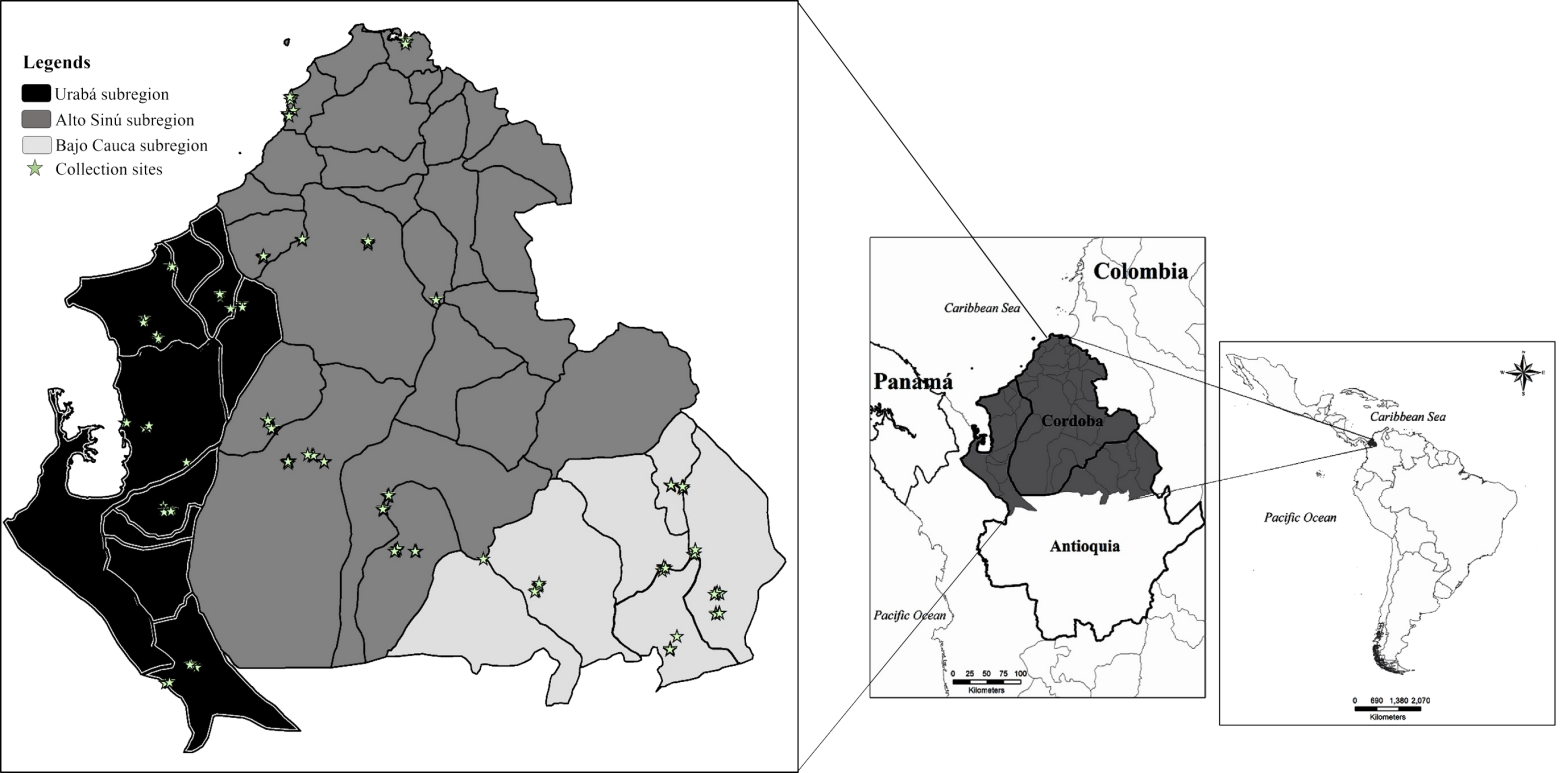
Study area and collection sites for *Anopheles nuneztovari*, *Anopheles darlingi*, and *Anopheles albimanus* across the Urabá-Bajo Cauca and Alto Sinú endemic region (UCS), Colombia.

Malaria is an infectious disease caused by protozoans of the genus *Plasmodium*; it is transmitted to humans by bites of female mosquitoes of the genus *Anopheles* [1]. In Latin America, Colombia occupies the third place in the number of malaria cases after Brazil and Venezuela, with 10% of the total number of reported cases [2]. In particular, the Urabá-Bajo Cauca and Alto Sinú (UCS, Fig 1) regions have often reported the highest numbers of malaria cases in Colombia [3]; only recently, in 2015, this area dropped to second in number, with 16.6% of total cases in the country [4]. For decades, *Plasmodium vivax* has been the predominant malaria parasite in UCS, representing an annual parasite index (API) of 28.7/1,000 inhabitants in 2015; while the API for *Plasmodium falciparum* was 10.2/1,000 [5]. The three main Colombian vectors *Anopheles darlingi*, *Anopheles nuneztovari* and *Anopheles albimanus* have an important role in malaria transmission in the UCS region [3,6,7].

Ecological niche models (ENMs) are used to understand ecological requirements of species, aspects of their biogeography, predict geographic distributions, identify sites for translocations and reintroductions, select areas for conservation and forecast effects of environmental change [8]. Studies of geographic distributions of vector species using ENM techniques relate occurrence records and environmental characteristics across species’ distributions [9–12]. For instance, ENM analyses of anopheline species (subgenus *Nyssorhynchus*) in Amazonian Brazil revealed diversification in habitat-use: *An*. *triannulatus* is a generalist, whereas *An*. *oryzalimentes* and *An*. *janconnae* are specialists [13]. ENMs were also used to predict distributions of *An*. *bellator*, *An*. *cruzii*, and *An*. *marajoara* of the Riviera Valley in southern Brazil, which revealed specific associations with land cover types [14]. Finally, low tolerance to dry environments was documented for *An*. *darlingi*; projected climate change would significantly reduce its suitable habitat mainly in Amazonian biomes, influencing both its distribution and abundance, in contrast to species of the Albitarsis complex [15].

Implementation of effective methods for ecological and epidemiological data analysis is essential in a country like Colombia, considering the rapid reduction of native habitats and their conversion to agriculture, livestock, and mining uses [16]. These changes make it a priority to identify variables that determine and constrain distributions of disease vector species. The limited resources available for regular monitoring of vector species and vector-borne diseases and control of infectious diseases would be improved by development of an early warning system regarding transmission and outbreaks of vector-borne diseases. Hence, we used ENM to evaluate potential vector distributions and to assess the hypothesis that environmental heterogeneity is a driver for allopatric distributions of possible competing niche-related species.

## Materials and methods

### Occurrence data

The endemic malaria region Urabá Bajo-Cauca Alto-Sinú (UCS) is located at northwest Colombian (Fig 1), has an estimated population at-risk of malaria equivalent to 2,500,000 people, distributed in the departments of Antioquia and Córdoba [17]. The predominant economic activities are mining, the banana agro-industry, cattle production and the timber industry; some of these activities have been linked to malaria transmission [3]. Twenty municipalities of UCS were sampled (Fig 1) between December 2012 and March 2015, using human landing-catches, under an informed consent agreement and collection protocol reviewed and approved by the Universidad de Antioquia Institutional Review Board (Bioethics Committee, Facultad Nacional de Salud Pública-Universidad de Antioquia, Acta 063). In light of the risk that these vectors represent for the transmission of the disease to humans, collections were performed in or near human residences. Two localities were selected per municipality (Table 1), based on criteria such as high numbers of reported malaria cases in 2012 and accessibility. Collections were conducted during three nights at each locality, by a team of four people, two persons sampling one site each night, for a total of six sites by locality, from 18:00 to 24:00 h, both indoors and outdoors. Adult mosquitoes were identified using a morphological key [18]; species assignation for a percentage of the specimens and all those presenting problems in morphological identification were confirmed by PCR-RFLP-ITS2 [19–21] and *COI* barcoding [22–24].

**Table 1:** Summary of mosquito collection data at 40 localities of Urabá-Bajo Cauca and Alto Sinú region, Colombia.

Subregion	Locality	Village	Species present	Date	Longitude, latitude	
Bajo Cauca	** **	** **	** **	** **	** **	** **
	Zaragoza	El Retiro	*An*. *nuneztovari*, *An*. *darlingi*	14-Dec-12	-74.8713889	7.4164444
		San Antonio	*An*. *nuneztovari*, *An*. *darlingi*	17-Dec-12	-74.8480556	7.4571111
	El Bagre	La Lucha	*An*. *nuneztovari*, *An*. *darlingi*	13-Oct-13	-74.7024167	7.5959167
		Villa Grande	*An*. *nuneztovari*, *An*. *darlingi*	15-Oct-13	-74.7046111	7.5333611
	Nechí	La Concha	*An*. *darlingi*	21-Oct-13	-74.8693889	7.9507222
		Puerto Astilla	*An*. *nuneztovari*, *An*. *darlingi*	18-Oct-13	-74.8270556	7.9440833
	Caucasia	Cuturú	*An*. *nuneztovari*, *An*. *darlingi*	20-May-14	-74.7887500	7.7250278
		Puerto Triana	*An*. *nuneztovari*, *An*. *darlingi*	17-May-14	-75.3223056	7.6200833
	Cáceres	Asturias	*An*. *nuneztovari*, *An*. *darlingi*	21-May-14	-75.3198333	7.6293611
		Campanario	none	25-May-14	-75.2315556	7.5840833
	Tarazá	El Rayo	none	5-Marz-15	-75.3678333	7.5309444
		Santa Clara	*An*. *nuneztovari*, *An*. *albimanus*	2-Marz-15	-75.5100833	7.7088889
Urabá			* *			
	Necoclí	Limoncito	*An*. *nuneztovari*, *An*. *albimanus*	27-Feb-13	-76.6660278	8.4911111
		Villa Sonia	*An*. *nuneztovari*, *An*. *darlingi*, *An*. *albimanus*	2-Marz-13	-76.6206389	8.4311111
	Arboletes	Naranjitas	*An*. *albimanus*	5-Marz-13	-76.3325278	8.5283611
		La Arenosa	*An*. *albimanus*	7-Marz-13	-76.4056389	8.5717778
	Mutatá	Bejuquillo	*An*. *nuneztovari*, *An*. *albimanus*	26-Feb-14	-76.5055278	7.3654722
		La Secreta	*An*. *nuneztovari*, *An*. *darlingi*	1-Marz-14	-76.5943889	7.3081944
	Apartadó	La Victoria	*An*. *nuneztovari*, *An*. *darlingi*, *An*. *albimanus*	4-Marz-14	-76.5768611	7.8689444
		Salsipuedes	*An*. *nuneztovari*, *An*. *albimanus*	8-Marz-14	-76.6021944	7.8833056
	Turbo	Camerún	*An*. *albimanus*	19-Nov-14	-76.7279722	8.1520556
		La Playona	*An*. *nuneztovari*, *An*. *albimanus*	16-Nov-14	-76.6548611	8.1343333
	San Juan de Urabá	Filo de Damaquiel	*An*. *albimanus*	21-Nov-14	-76.5803333	8.6609722
		La Mugrosa	none	24-Nov-14	-76.5495278	8.6663056
Alto Sinú						
	San Carlos	Sierra Chiquita	*An*. *albimanus*	8-Jun-13	-75.9031944	8.7387778
		Guacharacal	*An*. *darlingi*, *An*. *albimanus*	5-Jun-13	-75.6704444	8.5538056
	Valencia	San Rafael de Pirú	*An*. *nuneztovari*, *An*. *darlingi*	15-Jun-13	-76.2464722	8.1591667
		Santafe de Pirú	*An*. *nuneztovari*, *An*. *darlingi*	13-Jun-13	-76.2299167	8.1340833
	Moñitos	Broqueles	*An*. *nuneztovari*, *An*. *albimanus*	7-Nov-13	-76.1662222	9.2158056
		Rio Cedro	*An*. *albimanus*	9-Nov-13	-76.1743889	9.1534444
	San Antero	Bahía Cispatá	*An*. *albimanus*	13-Nov-13	-75.7815833	9.3946944
		Tijereta	none	14-Nov-13	-75.7881944	9.2920833
	Montelibano	Puerto Anchica	*An*. *nuneztovari*, *An*. *darlingi*, *An*. *albimanus*	18-Jul-14	-75.8498333	7.8743333
		Puerto Nuevo	*An*. *nuneztovari*, *An*. *albimanus*	22-Jul-14	-75.8333333	7.9153333
	Puerto Libertador	La Piedra	*An*. *nuneztovari*	23-Jul-14	-75.8074444	7.7353889
		Villanueva	*An*. *darlingi*	28-Jul-14	-75.7405556	7.8350556
	Canalete	Buenos Aires de las Pavas	*An*. *albimanus*	2-Nov-14	-76.1278889	8.7488889
		El limon	*An*. *albimanus*	4-Nov-14	-76.2606944	8.6889167
	Tierralta	Tuistuis Arriba	*An*. *nuneztovari*	6-Nov-14	-76.0899444	8.0431667
		Santa Ana	*An*. *nuneztovari*, *An*. *albimanus*	8-Nov-14	-76.1754167	8.0195278

### Environmental variables

To characterize environmental variation across the study region, the Normalized Difference Vegetation Index (NDVI) was used. NDVI is a measure of photosynthetic activity; its variation reflects the spatial and temporal dynamics of vegetation that indirectly influence mosquito reproduction and development [25]. NDVI data were obtained in the form of imagery from the Moderate Resolution Imaging Spectroradiometer (MODIS) Terra satellite, at 250 m spatial resolution and 16-day temporal resolution (*https*:*//reverb*.*echo*.*nasa*.*gov/reverb/*). In all, 69 images from 2012 to 2014 were used. Images were reprojected to the MAGNA-SIRGAS coordinate system using the MODIS Reprojection Tool [26]. To reduce inter-correlation among data layers, a principal components analysis (PCA) was performed using all 69 NDVI images as variables [27]. Climatic information was not included, owing to the coarse spatial resolution of such data sets, which would not permit fine-grained predictions. Regardless, rainfall is indeed reflected in the NDVI data via vegetational responses and consequently increased or decreased photosynthetic mass [28].

### Ecological niche modeling

To evaluate potential distributions of the vector species, ENMs were developed using a maximum entropy approach implemented in Maxent, version 3.3.3k [29]. Maxent assesses suitability for species by integrating occurrence records with relevant environmental predictors across a defined geographic space [29]. Maxent attempts to estimate a target probability distribution by finding the probability distribution of maximum entropy (i.e., that which is most spread out, or closest to uniform), subject to a set of constraints that represent the incomplete nature of information about the target distribution [30]. A sampling bias layer was designed to improve niche and range estimates with Maxent and to point process models by integrating spatially explicit information [30], with our sampling locations defined as 1; the remaining pixels that were not sampled had “no data” in the grid. Given the broad potential distribution previously reported for *An*. *nuneztovari*, *An*. *albimanus*, and *An*. *darlingi* in South America [15,31,32] and the known distribution of the species across the country [3,7,18,21], a very broad hypothesis of accessible area (**M**) was used, considering the entire endemic region as accessible [33].

To assess how robust and predictive the models were, occurrence data for each species were split into training (50%) and testing (50%) subsets; this random splitting was done five times. No clamping or extrapolation was permitted in MaxEnt; remaining parameters were left as default. Logistic output formats were used. ENMs were calibrated for each species, with 10 bootstrapped replicates; the median across replicates was used as a basis for further analysis. All maps were converted to binary via a conservative least presence thresholding approach, consisting of the lowest predicted value corresponding to any occurrence record of the species in the calibration data set [34,35].

The partial receiver operating characteristic (ROC) approach was used to assess models performance [36]; the evaluation dataset was bootstrapped, and probabilities obtained by direct count of area under the curve (AUC) ratios falling ≤ 1 via a Visual Basic script (N. Barve, pers.comm.; https://kuscholarworks.ku.edu), with 100 iterations. Model performance was tested for different combinations of principal components (the first 5, 10, 15, 20, 25, 30, 35, 40, 45, 50, and 55 components). The omission rate and partial ROC AUC ratios were used as criteria to select optimal environmental data sets for each species. Final models were produced for each species using all available data [37]. We inspected the loading values of each raw variable (16-day composite NDVI) on each of the first two principal components and how they related to monthly rainfall averages in the study area to have a better understanding of how vegetation dynamics reflected in NDVI related to suitability for each species. We also compared available (i.e., within **M**) and occupied environments in terms of NDVI values across the endemic area. The occupied niche was represented in two ways by: (1) considering NDVI values over the study period for each occurrence coordinate, and (2) taking only NDVI values matching the date of the mosquito’s capture.

### Niche overlap

To evaluate a hypothesis of niche similarity or difference among the three species within this endemic area, we used a background similarity test implemented in ENMTools v.1.4.3 [38]. The test generates a similarity measure by overlaying predictions of the two species compared [38]; niche overlap values were calculated using the Schoener’s *D* metric, with values ranging from 0 (no overlap) to 1 (complete overlap). The background similarity test uses the original predictions for each species and compares each of them with overlaps generated from models based on random occurrences selected from across the accesible area for each species. Observed similarity was compared with the distribution of similarities between the focal species and the suite of null models. Probabilities were determined by direct count, and two comparisons were made for each pair of species, with each species serving in turn as the focal species. In all, 100 pseudoreplicates were generated for each pair [38,39]. Finally NicheA was used to visualize overall overlap based on minimum volume ellipsoids for the species in three PCA dimensions [40]; the Jaccard index (*I*_*J*_) was used as a numerical estimation of environmental overlap among species [41].

## Results

Presence records totaled 100 for *Anopheles nuneztovari*, 69 for *Anopheles darlingi*, and 88 for *Anopheles albimanus*. All models developed performed statistically significantly better than random expectations (all *p*<0.01; S1 Table). According to omission rate and ROC partial values, optimal principal component combinations were, *An*. *nuneztovari* first 25 PCAs, *An*. *darlingi* first 35 PCAs, and *An*. *albimanus* first 25 PCAs.

**Fig 2:**
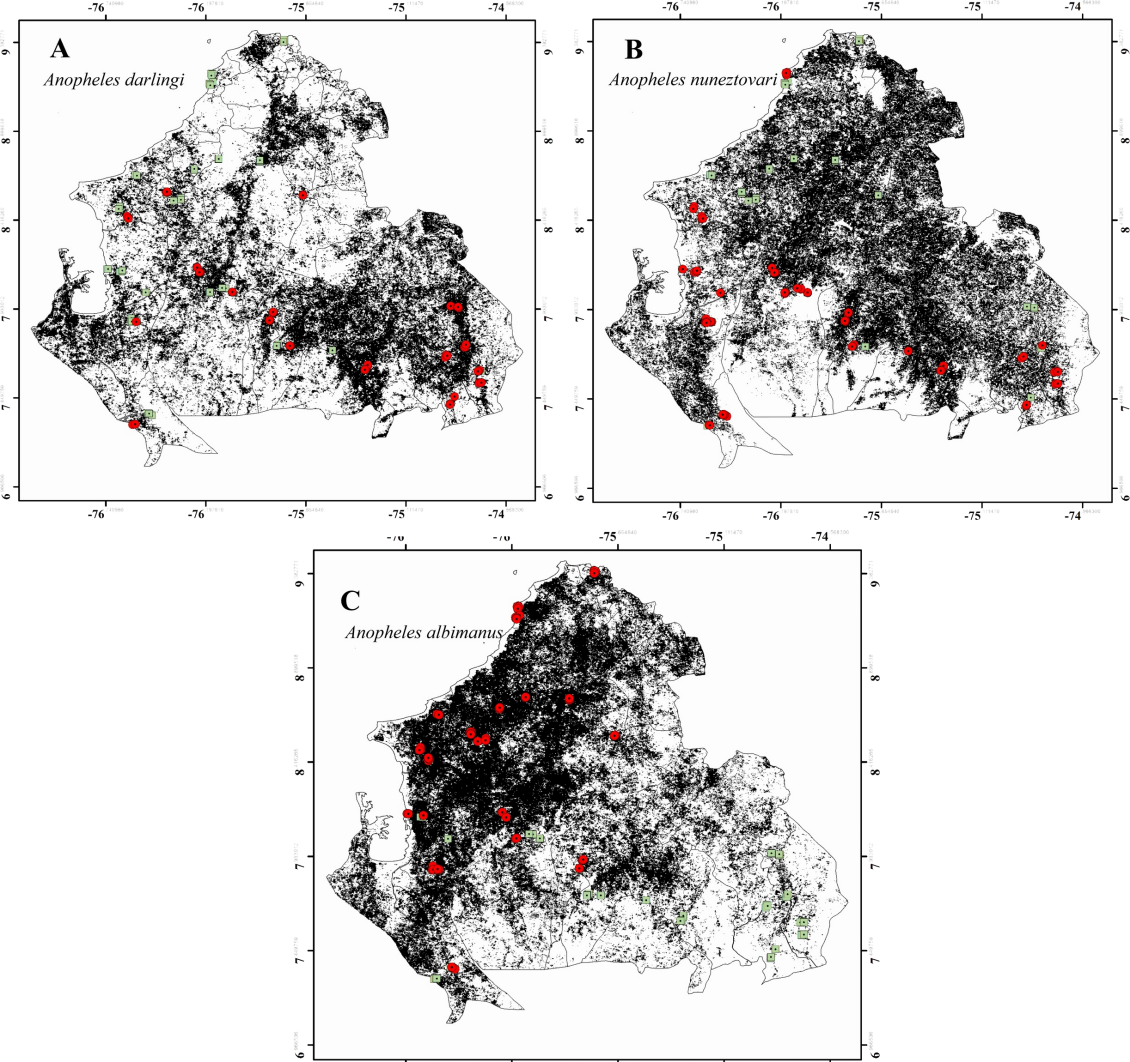
Ecological niche models. Showing environmental suitability for the three Colombian main malaria vectors across the Urabá-Bajo Cauca and Alto Sinú endemic region. Red circles indicate location of the records used for model calibration and green squares indicate absence records.

Potential distributions estimated for the three species indicate that the area with highest environmental suitability for *An*. *darlingi* was Bajo Cauca (39% suitable; Fig 2A). In contrast, the broadest potential distribution for *An*. *nuneztovari* was in parts of Bajo Cauca and Alto Sinú (48.3% suitable; Fig 2B). The most suitable areas for *An*. *albimanus* were in coastal areas of Urabá and Alto Sinú and some areas of Bajo Cauca (31% suitable; Fig 2C).

**Fig 3:**
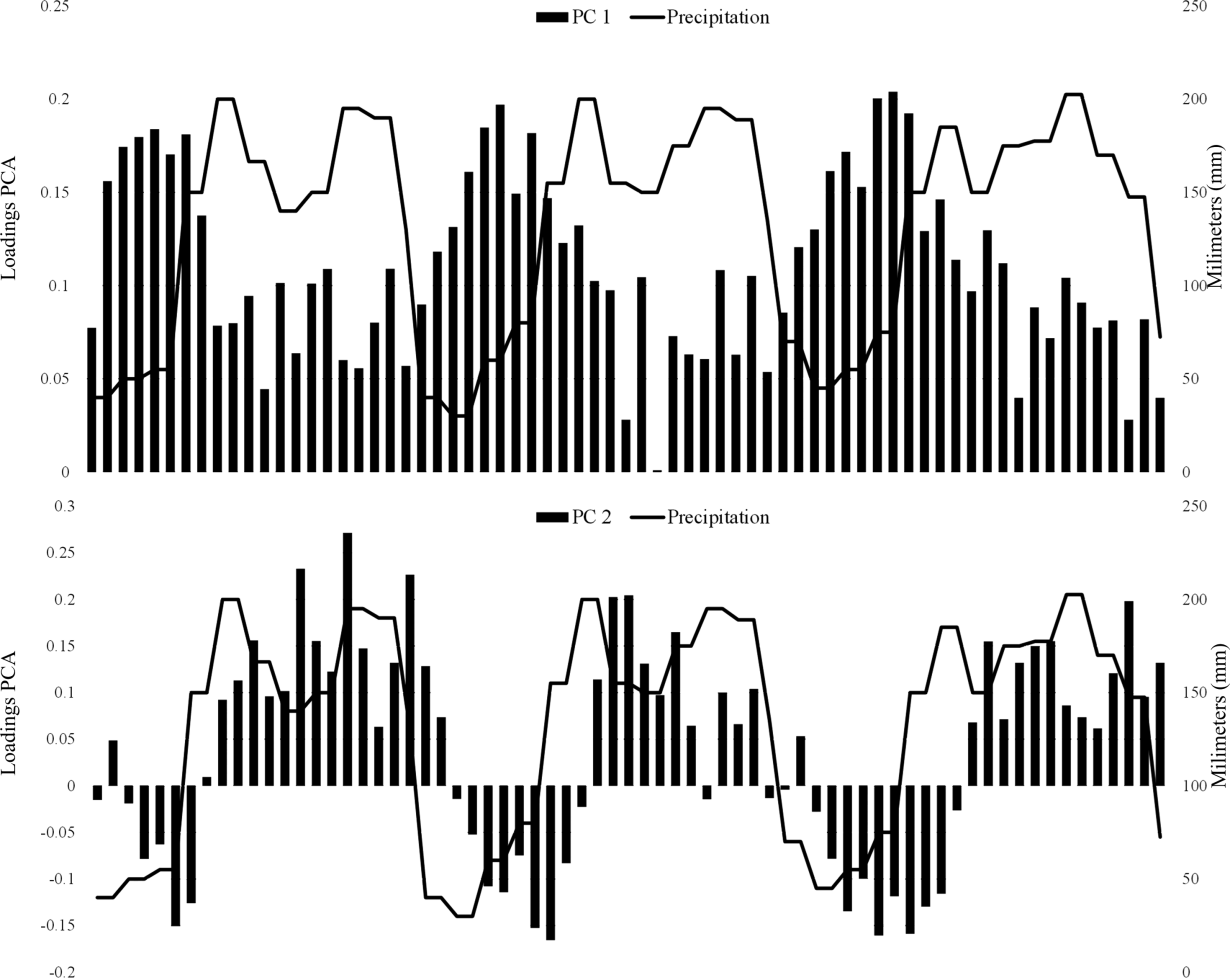
Loading plot from a principal component analysis using NDVI values for the Urabá-Bajo Cauca and Alto Sinú region of Colombia. Black bars indicate the relationship (positive or negative) of the principal components with the dates of the NDVI values. Black line represents the average monthly rainfall for the study area (data from Instituto de Hidrología, Meteorología y Estudios Ambientales de Colombia IDEAM, Octubre 2016).

**Fig 4:**
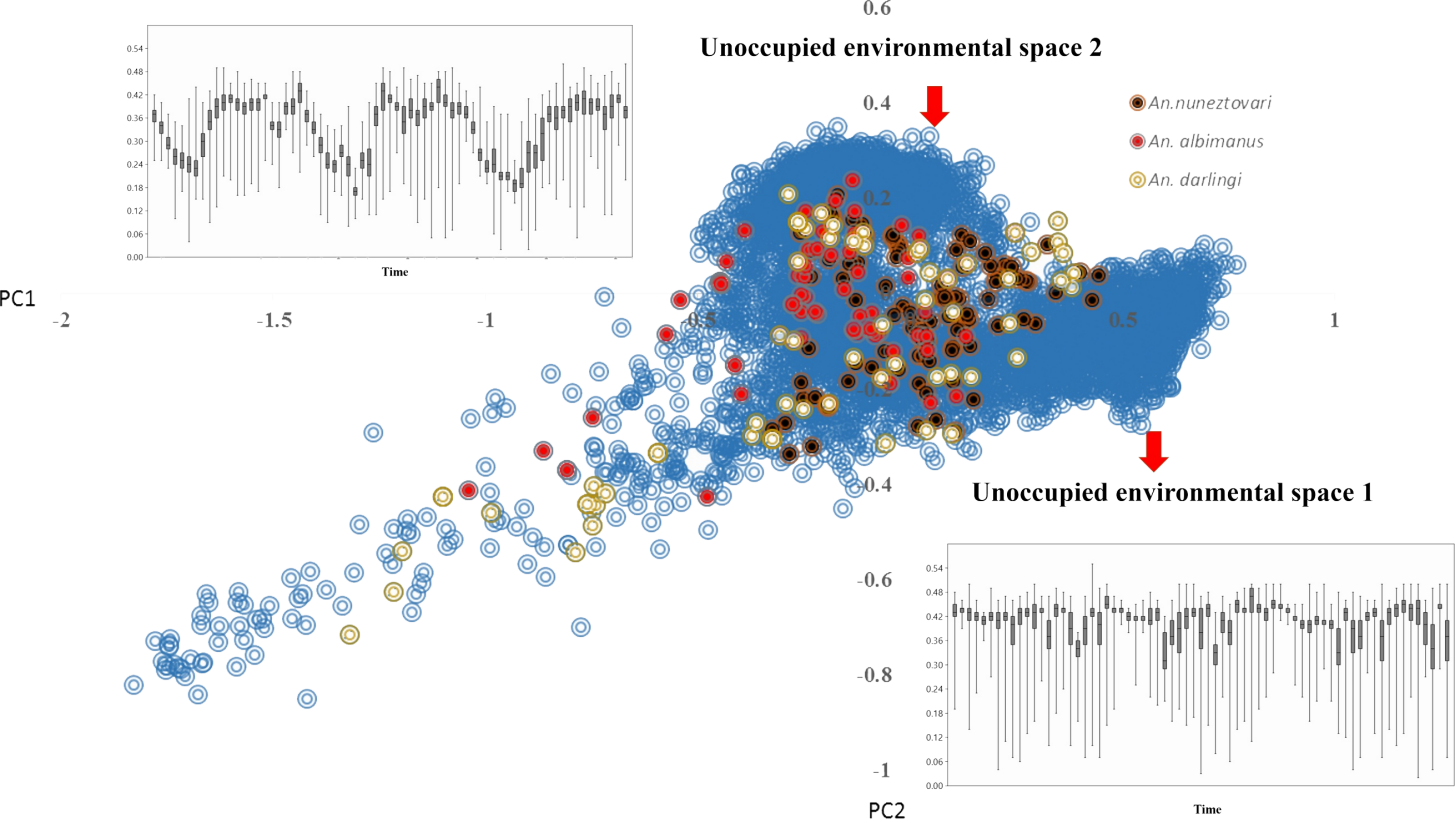
Results of the principal component analysis. Based on NDVI values for the Urabá-Bajo Cauca and Alto Sinú region of Colombia. Blue circles indicate NDVI values for sites across the study area. Other colored circles represent NDVI values for occurrence data for the main malaria vectors *An*. *nuneztovari*, *An*. *albimanus*, and *An*. *darlingi*. Red arrows indicate regions of available, but unoccupied environmental space. Box plots represent NDVI values in those unoccupied areas of environmental space (1 and 2).

**Fig 5:**
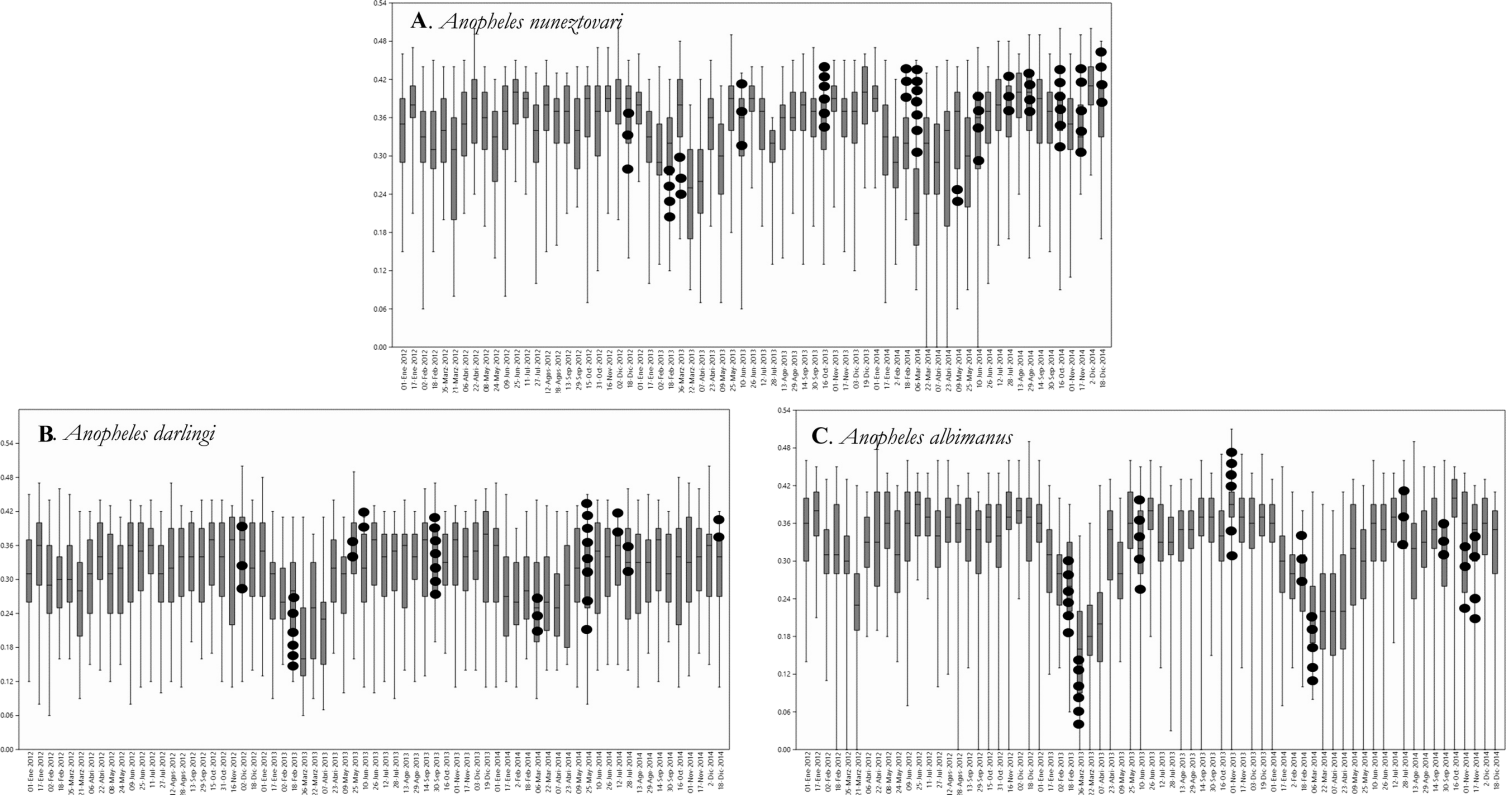
NDVI values at all occurrence sites for the three malaria vectors in northern Colombia. The black dots represent specific NDVI values corresponding to mosquito collection dates.

The relationship between the original variables (NDVI values) and the principal components showed that the first principal component (PC1) was positively associated with NDVI for all dates throughout the three years, while PC2 exhibited positive and negative associations with NDVI in each of the three years. Both PC1 and PC2 exhibited temporal trends associated with NDVI variation, reflecting seasonality and corresponding leaf phenology, partially in response to fluctuation of rain (Fig 3), which is evident in two areas that are unoccupied and that present contrasting seasonality, was well as overall levels of NDVI (Fig 4). Areas with highest PC1 values had high NDVI values and relatively little seasonal fluctuation, whereas areas with the highest PC2 values had clear fluctuations in both years. PC1 and PC2 explained 39.7% and 11.0% of the total variance, respectively. *Anopheles albimanus* and *An*. *darlingi* occupied areas with lower NDVI values throughout the year (Fig 5) as compered than *An*. *nuneztovari*. All species were apparently absent from areas with high NDVI values throughout the year (evergreen forests) and areas with very pronounced NDVI fluctuations.

**Fig 6:**
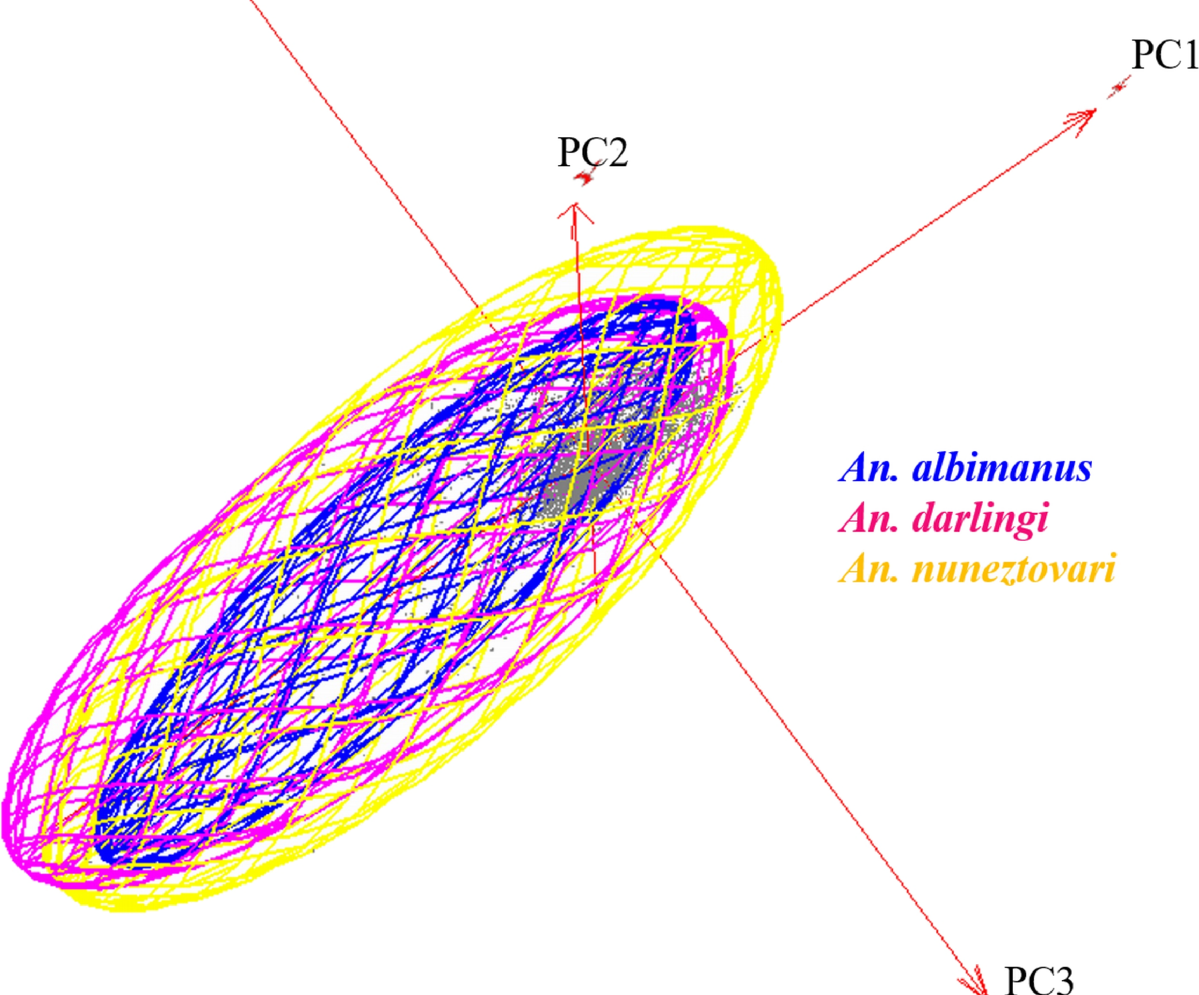
Environmental space modeled in NicheA. Visualization in three dimensions in terms of principal components 1, 2 and 3; ecological niche models were displayed as minimum-volume ellipsoids used to illustrate limits of environmental distributions. The background cloud (in gray), was derived from a random sample of 3000 random points from across the study area.

Assessment of both geographic and environmental dimensions showed high overlap among the three species (*D* > 0.35). Nonetheless, observed overlap between *An*. *nuneztovari* and *An*. *albimanus* was significantly lower than expected from a null distribution (*p* = 0.032 in the background similarity tests, S1 Fig). NicheA visualization revealed broad overlap among the niches of the three species, and showed that niche breadth was greater for *An*. *nuneztovari* than for the other two species (Fig 6). The Jaccard index indicated that environmental overlap between *An*. *nuneztovari* and *An*. *darlingi* was 0.63, that for *An*. *nuneztovari* versus *An*. *albimanus* was 0.36, and that for *An*. *albimanus* versus *An*. *darlingi* was 0.13. These results are consistent with Schoener’s *D* metric.

## Discussion

The three main Colombian *Anopheles* vector species, *An*. *nuneztovari*, *An*. *albimanus*, and *An*. *darlingi*, occur in sympatry in the Urabá-Bajo Cauca and Alto Sinú region [3,7,42]. Using remotely sensed environmental data with high spatial and temporal resolution, we were able to identify small but meaningful differences in environments occupied by these three species. These details can improve the utility of ENMs with the aim of improving the knowledge of the biology of these important species, and guiding integrated vector management efforts [43]. Deforestation and changes in land use are occurring at a higher speed than changes in climate; as a result, use of environmental variables indicative of changes in vegetation cover will be a useful input in the prediction and prevention of vector-borne disease transmission.

These results agree with previous efforts to map *Anopheles* species’ distributions in the Americas, in which models indicated their presence across our study region [12,15,31,32]. At difference from those studies, we used environmental layers with higher spatial and temporal resolution and thus greater information content at finer scales, allowing documentation of more specific and detailed distributional patterns. The three main Colombian vectors *An*. *darlingi*, *An*. *nuneztovari* and *An*. *albimanus* have an important role in malaria transmission in the UCS region [3,6,7]. *Anopheles nuneztovari* and *An*. *darlingi*, have been reported naturally infected by *Plasmodium* spp. in UCS [8], and reports have indicated that these two species are able to maintain transmission even at low abundances [4]. While *An*. *albimanus* has a predominant coastal distribution and in the Colombian Pacific Coast was detected infected with *Plasmodium vivax and Plasmodium falciparum* [6]. Our final results showed that *An*. *nuneztovari* has the widest geographic distribution, occupying almost the entire study region; this breadth is probably related to the ability of this species to colonize both natural and disturbed environments [3,44]. *Anopheles nuneztovari* is the most frequently detected species in some localities of the Bajo Cauca and Alto Sinú region [3,7]. *Anopheles darlingi* is also an important malaria vector in other regions of Colombia [45,46], it has been associated with breeding sites in riverine or gallery forest [47–50]. Our model for *An*. *darlingi* showed that most suitable localities for this species are in Bajo Cauca; specifically, along the Cauca and Nechí rivers, that conform an important aquifer system in this region [51]. The riparian ecosystems in this subregion and the potential for rapid adaptation by this species to novel environments [49,52,53] may favor the establishment of populations of this species. Particularly, *An*. *darlingi* is associated with riparian ecosystems in the upper Orinoco River in southern Venezuela, where overflow of the river creates lagoons that constitute suitable larval habitats [54]. Therefore, the results of the present study suggest the importance of authorities enforcing regulations on deforestation for the Cauca and Nechí rivers basins, where illegal activities such as gold mining may be increasing human contact exposition to *An*. *darlingi*, as has been previously suggested [55].

Finally, in Colombia, *An*. *albimanus* is a species with a mostly coastal distribution [7,56,57]. The model identified suitable areas for *An*. *albimanus* in Urabá and Bajo Cauca. Absence of this species from some areas modeled as suitable may relate to factors such as competitive exclusion, existing vector control measures, or rapid changes in land use as a result of agricultural activities and mining, as previously suggested for this species in Mesoamerica and the Caribbean region [32].

Previous studies have shown that distributions of mosquito species are partly related to land use factors such as presence or absence of wetlands, type of surrounding vegetation, and agricultural practices [58]. Various studies on species modeling are using NDVI instead of land use factors, given that this index is a measure of photosynthetic activity; thus, allows an indirect approximation to the suitable conditions for mosquito development [25]. Our result concerning the relationship between precipitation and NDVI patterns in different time periods showed that PC1 was more indicative of overall NDVI values (ie; forest versus non-forest), whereas PC2 was more indicative of the magnitude of seasonality in NDVI. Apparently, the three main Colombian *Anopheles* vector species in Urabá-Bajo Cauca and Alto Sinú do not occupy environments either with high seasonality, or with low seasonality and high NDVI. Because NDVI has been used to estimate vegetation characteristics [59], the relation between NDVI and species’ occurrence suggests similar affinities of the three species for particular vegetation types; *An*. *albimanus* tended to occupy places with relatively low NDVI values, representing ecosystems with bare soils and low forest cover [60]. Previous reports indicate that *An*. *albimanus* is associated with larval habitats exposed to sun [48], which as in the present study, are related to scarce forest cover, and also its larvae can tolerate some salinity [61]. Places with low seasonality and high NDVI values probably represent residual forests unoccupied by people.

The background similarity tests and NicheA analysis showed high overlaps in the geographic and environmental conditions occupied by *An*. *darlingi*, *An*. *nuneztovari* and *An*. *albimanus*. The dispersal capacity of these species and their ability to colonize different ecosystems are well documented [3,53,62,63]. These aspects may facilitate their sympatry across areas presenting diverse environmental conditions.

The models developed in this study have important applications, since they could be projected or replicated for different eco-epidemiological malaria zones of Colombia, as has been done elsewhere in the world [64]. In other applications of these approaches, models of the potential geographic distribution of Ebola and Marburg viruses in Africa [65] were able to anticipate the potential for Marburg outbreaks in Angola [66]. Variation in NDVI in relation to the potential distribution of the vector species may be reflected in patterns of malaria transmission in Urabá-Bajo Cauca and Alto Sinú region. Historically and until recently, this region reported the highest numbers of malaria cases in Colombia [

3]. The main vectors *An*. *nuneztovari* and *An*. *darlingi* have been detected naturally infected by *Plasmodium* spp. in UCS [7], and recent reports indicate that these species are the most important malaria vectors in some localities of the region [3]. Vector distribution and malaria transmission might be highly dynamic because of antrophic or natural causes; thus a good characterization of environmental variation, through the use of NDVI at high spatio-temporal resolution [58,67,68] to predict mosquito spreading, will provide baseline information that can be used to reduce malaria risk in this region.

## Supporting information

S1 Table Partial AUC ratios of *Anopheles* vector species. (DOCX)

S1 Fig Background similarity test. (PDF)
