# Supplementary material for: Potential distribution of mosquito vector species in a primary malaria endemic region of Colombia
Source: PLoS One. 2017 Jun 8;12(6):e0179093. doi: 10.1371/journal.pone.0179093 (PMC5464628; doi:10.1371/journal.pone.0179093)
Supplement: S1 Table — (DOCX) [file pone.0179093.s001.docx]

S1. Table. Partial AUC ratios of *Anopheles* vector species ecological niche models based on Normalized Difference Vegetation Index (NDVI)

| Species | Mean AUC ratio | Probability |
| --- | --- | --- |
| *Anopheles albimanus* | 1.5677 | 2.20E-16 |
| *Anopheles darlingi* | 1.2748 | 1.62E-13 |
| *Anopheles nuneztovari* | 1.3057 | 1.15E-13 |
