# Supplementary material for: Potential distribution of mosquito vector species in a primary malaria endemic region of Colombia
Source: PLoS One. 2017 Jun 8;12(6):e0179093. doi: 10.1371/journal.pone.0179093 (PMC5464628; doi:10.1371/journal.pone.0179093)
Supplement: S1 Fig — (PDF) [file pone.0179093.s002.pdf]

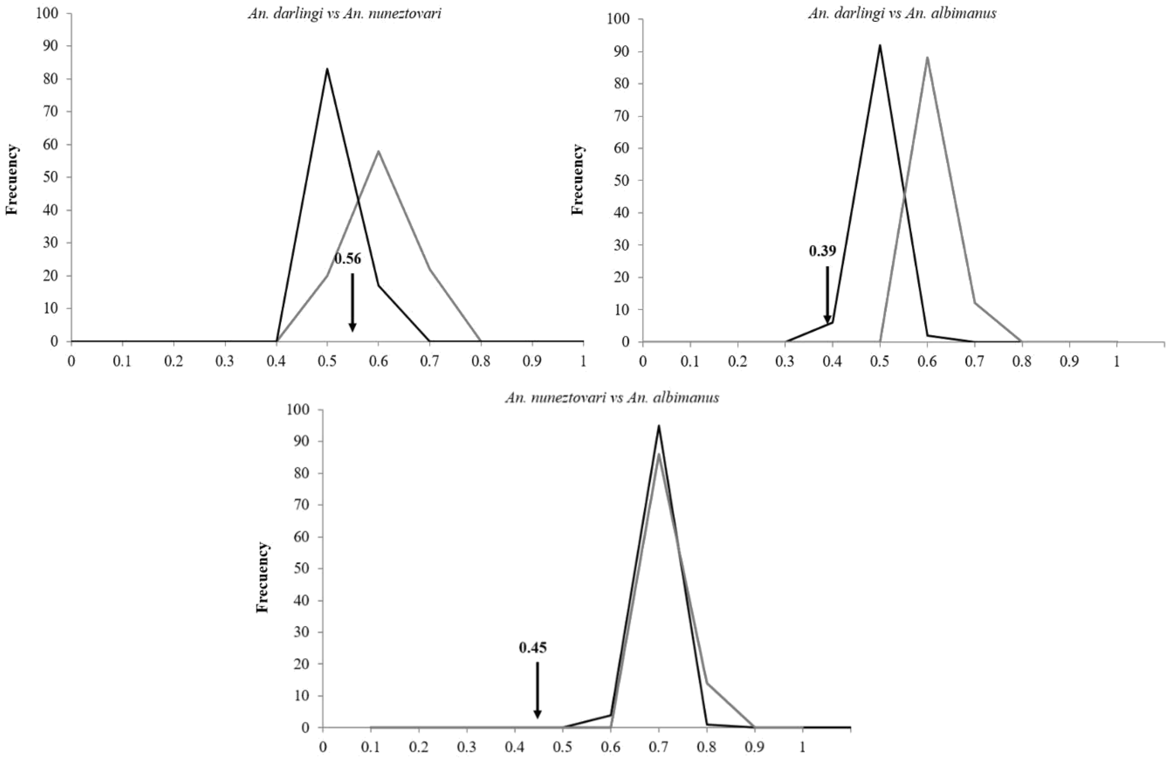

S1 Fig. Background similarity test. Lines represent the frequency of the values of the D index from 100 null models generated from random points (unit A vs. unit B, gray line; and unit B vs. Unit A, black line). Arrows indicate the observed D value in the overlap test (truly observed value between units A and B). Overlap values falling in or below the lower left 5% of the null distribution are interpreted as indicative of significant niche divergence.
